# Interpretable machine-learning prediction of severe myelosuppression in colorectal cancer patients receiving chemotherapy using XGBoost and SHAP: a retrospective study with a web-based calculator

**DOI:** 10.3389/fonc.2026.1785146

**Published:** 2026-03-19

**Authors:** Linxian Ding, Lixia Peng, Zheng Xu, Zhangli Cui, Zhongming Wang

**Affiliations:** 1 Department of Oncology, Shidong Hospital, Yangpu District, Shidong Hospital Affiliated to University of Shanghai for Science and Technology, Shanghai, China; 2 College of Mathematics, Beijing Normal University, Beijing, China

**Keywords:** chemotherapy, colorectal cancer, extreme gradient boosting, interpretability, prediction model, severe myelosuppression

## Abstract

**Background:**

Patients with colorectal cancer (CRC) are susceptible to severe myelosuppression (SMS) after chemotherapy. Conventional linear models may have limited performance and may fail to capture complex, nonlinear risk patterns, which can hinder early risk stratification and timely intervention. We aimed to develop an interpretable machine-learning model to predict SMS and to build a web-based calculator for individualized risk assessment.

**Methods:**

We retrospectively enrolled 987 CRC patients who received capecitabine plus oxaliplatin with or without targeted therapy at our hospital between March 2021 and November 2025. Nine predictors were selected using least absolute shrinkage and selection operator (LASSO) regression. We developed and compared several models, including extreme gradient boosting (XGBoost), random forest, decision tree, and support vector machine. Model interpretability was assessed using SHapley Additive exPlanations (SHAP) at both the global and individual levels to characterize nonlinear effects and feature interactions. A web-based, real-time risk calculator was also implemented.

**Results:**

On the validation set, the XGBoost model achieved the best balance of predictive performance (AUC = 0.906; sensitivity = 0.864). SHAP analysis quantified the contribution of each feature, with the top three contributors being white blood cell count, number of chemotherapy cycles, and Karnofsky Performance Status score. Nonlinear threshold effects were observed for continuous variables, including white blood cell count, platelet count, and serum albumin. Interactions were identified between white blood cell count and performance status, as well as between white blood cell count and number of chemotherapy cycles. The web-based calculator enables real-time individualized risk estimation. Decision curve analysis indicated favorable net clinical benefit across a range of decision thresholds.

**Conclusion:**

We developed a high-performing and interpretable model for predicting SMS in CRC patients receiving chemotherapy. The accompanying web-based calculator may provide a practical tool for early risk stratification and individualized management of chemotherapy-related SMS.

## 1 Introduction

Colorectal cancer (CRC) is a common malignancy in China. Patients with early-stage disease are primarily treated with surgery followed by adjuvant chemotherapy, whereas those with advanced disease often require systemic therapy, including chemotherapy combined with targeted therapy or immunotherapy (1, 2). However, chemotherapy-induced myelosuppression is among the most frequent and dose-limiting toxicities in CRC. It may present as neutropenia, thrombocytopenia, and anemia; in severe cases, it can lead to infections or bleeding, resulting in treatment delay, dose reduction, or discontinuation, and potentially compromising antitumor efficacy and survival (3, 4). Given that many CRC patients receive multi-agent, multi-cycle regimens, the burden of severe myelosuppression (SMS) is substantial (5, 6). Previous studies have reported that the incidence of grade III~IV myelosuppression ranges from 20.4% to 47% in patients with colorectal cancer undergoing chemotherapy, depending on the regimen and patient population (7–9). In routine practice, risk management largely relies on periodic blood count monitoring, which may not identify high-risk patients early enough to enable timely preventive interventions (10, 11). Therefore, an accurate prediction model for SMS is needed to support early risk stratification and individualized management.

In recent years, several studies have investigated risk factors for SMS in different cancer populations. Most have used univariate screening followed by multivariable logistic regression and, in some cases, nomograms for clinical prediction (12–14). However, in real-world datasets, SMS is often driven by complex, nonlinear relationships and interactions among predictors. Traditional regression models rely on linearity and additivity assumptions and may fail to capture these patterns, potentially limiting predictive performance (15, 16). In addition, many existing models provide limited individual-level interpretability, making it difficult to clearly quantify how each feature contributes to a specific patient’s predicted risk and thereby constraining their use in personalized decision-making.

With the increasing adoption of machine learning in healthcare, ensemble algorithms such as extreme gradient boosting (XGBoost) have demonstrated advantages in modeling high-dimensional data and capturing nonlinear relationships (17, 18). Meanwhile, interpretability approaches such as SHapley Additive exPlanations (SHAP) have enabled transparent, explainable predictions without substantially sacrificing performance (19, 20). Therefore, we developed an SMS risk prediction model for CRC patients receiving capecitabine plus oxaliplatin with or without targeted therapy (CapeOx ± Targ) using XGBoost to address the limitations of traditional regression models. We applied SHAP for both global and individual-level interpretability to quantify feature contributions, visualize key risk drivers, and explore nonlinear and interaction effects. Finally, we implemented the model as a web-based, real-time risk calculator to support intuitive, individualized risk assessment and clinical decision-making during chemotherapy.

## 2 Methods

### 2.1 Study population and grouping

Patients with CRC who received chemotherapy at our hospital between March 2021 and November 2025 were retrospectively enrolled. The inclusion criteria were as follows: (1) pathologically or cytologically confirmed diagnosis of CRC; (2) receipt of a chemotherapy regimen consisting of CapeOx with or without targeted therapy (bevacizumab or cetuximab), administered according to National Comprehensive Cancer Network (NCCN) guidelines (21); (3) completion of at least one prior chemotherapy cycle; (4) age ≥18 years; and (5) availability of complete clinical data.

The exclusion criteria were: (1) presence of other malignant tumors; (2) presence of autoimmune diseases; (3) chemotherapy dose intensity <75% of the standard dose; and (4) concurrent use of medications known to affect bone marrow function (e.g., methotrexate, valproic acid, carbamazepine, interferon−α, or long-term systemic corticosteroids).

According to the Common Terminology Criteria for Adverse Events (CTCAE) version 5.0, SMS was defined as the occurrence of grade III~IV hematologic toxicity following the evaluated chemotherapy cycle, based on the following laboratory thresholds: hemoglobin < 80 g/L, WBC count < 2.0 × 10^9^/L, or PLT count < 50.0 × 10^9^/L. Patients who developed SMS were classified into the SMS group, whereas those with no myelosuppression or grade I~II myelosuppression were classified into the non-SMS group. In this study, for each included cycle, the predictors were derived from clinical and laboratory data collected within 14 days prior to that cycle, reflecting the patient’s status after the previous cycle, and the outcome was defined as the occurrence of SMS following that same cycle.

### 2.2 Sample size estimation

The sample size was estimated using a method described in the literature (22). Based on a preliminary review of medical records at our hospital, the incidence of SMS was estimated to be approximately 30%. With the significance level (α) set at 0.05 and the allowable error (δ) set at 0.05, the minimum required sample size was calculated as 323 patients for the training set and 139 patients for the validation set.

### 2.3 Data collection

Based on a literature review and expert consultation, a total of 29 potential predictors of SMS were initially considered. Clinical variables included age, sex, tumor location (right-sided colon vs. left-sided colon and rectum), tumor stage (stages I~III vs. stage IV), history of surgery, hypertension, diabetes mellitus, smoking status, bone metastasis, liver metastasis, lung metastasis, number of prior chemotherapy cycles before the current cycle, use of targeted therapy, Karnofsky Performance Status (KPS) score (categorized as ≤70 vs. ≥80), RAS mutation status, BRAF mutation status, and microsatellite instability status. Laboratory variables included C-reactive protein (CRP), pre-chemotherapy white blood cell count (WBC), pre-chemotherapy platelet count (PLT), pre-chemotherapy hemoglobin (Hb), serum albumin, creatinine, blood glucose, alanine aminotransferase (ALT), aspartate aminotransferase (AST), total bilirubin, D-dimer, and carcinoembryonic antigen (CEA). For each cycle, predictors were derived from clinical and laboratory data collected within 14 days prior to chemotherapy. For WBC, PLT, Hb, and serum albumin, the lowest value during this window was used; for the remaining laboratory variables (CRP, creatinine, glucose, ALT, AST, bilirubin, D-dimer, and CEA), the highest value was used.

### 2.4 Statistical methods

#### 2.4.1 Baseline characteristics and missing data handling

Baseline characteristics were analyzed using Stata version 18.0. Categorical variables were summarized as frequencies and percentages and were compared using the *χ²* test or Fisher’s exact test, as appropriate. Continuous variables were assessed for normality (Shapiro-Wilk test) and homogeneity of variance (Levene’s test). Depending on distribution, continuous variables were compared using the independent-samples *t* test or the Mann-Whitney *U* test. A two-sided *P* value <0.05 was considered statistically significant. The dataset contained a small proportion of missing values (overall missing rate <3%). To minimize potential bias from complete-case analysis, missing data were handled using multiple imputation with the mice package in R (version 4.5.0). Five imputed datasets were generated, and estimates were pooled according to Rubin’s rules. The dataset was split into training and validation sets before imputation, and imputation was performed separately within each set to avoid information leakage.

#### 2.4.2 Model development and evaluation

All model development and analyses were performed in R (version 4.5.0). Predictor selection was conducted using least absolute shrinkage and selection operator (LASSO) regression with the glmnet package. The optimal penalty parameter (λ) was determined using 10-fold cross-validation based on the minimum cross-validated deviance. Logistic regression was used as the baseline model. Four nonlinear machine-learning models—XGBoost, random forest, decision tree, and support vector machine (SVM)—were trained and compared with the baseline model. The XGBoost model was trained with the following hyperparameters: nrounds = 200, eta = 0.01, max_depth = 4, objective = “binary:logistic”, and eval_metric = “logloss”. To address class imbalance, the scale_pos_weight parameter was set as (number of negative cases/number of positive cases) × 2. Model performance was evaluated using accuracy, sensitivity, specificity, F1 score, and the area under the receiver operating characteristic curve (AUC).

#### 2.4.3 Interpretability analysis of features

Model interpretability was assessed using SHAP. SHAP values were calculated using the shapviz package in R (version 4.5.0). Global feature importance was visualized using bar plots, and beeswarm plots were generated to display the distribution of SHAP values across features. Individual-level explanations were illustrated using waterfall plots for representative cases. SHAP dependence plots were used to explore potential nonlinear effects and feature interactions.

#### 2.4.4 Development of a web-based SMS risk calculator

To facilitate clinical implementation, a web-based interactive application was developed using Python (version 3.11) and Streamlit (version 1.28). The backend of the calculator incorporated the trained XGBoost model, while the frontend provided an intuitive interface for clinicians to enter the nine predictors selected by LASSO regression. The calculator outputs the predicted probability of SMS and the corresponding risk classification in real time. The application followed a RESTful design, with data exchanged between the frontend and backend in JSON format to ensure real-time performance and stability. The system can be deployed on a server and accessed via a URL across multiple platforms.

#### 2.4.5 Model calibration and clinical net benefit evaluation

Calibration curves were plotted to assess agreement between predicted probabilities and observed outcomes. Decision curve analysis (DCA) was performed using the rmda package in R (version 4.5.0) to quantify the net benefit of the XGBoost model across a range of decision thresholds and to evaluate its potential clinical utility.

## 3 Results

### 3.1 General characteristics of the study cohort

A total of 1004 patients met the inclusion criteria, of whom 17 were excluded. Ultimately, 987 colorectal cancer patients receiving CapeOx ± Targ were included in the analysis. Among them, 306 patients experienced SMS, corresponding to an incidence of 31.0%. The cohort was randomly divided into a training set (691 patients, 70%) and a validation set (296 patients, 30%). Baseline characteristics of the study population are summarized in **Table 1**. The sample sizes for both the training and validation sets exceeded the calculated minimum required sample sizes. No statistically significant difference was found in SMS incidence between the training and validation sets (*P* = 0.571).

**Table 1:** Baseline characteristics of the study participants [M (P_25_, P_75_) or *n* (%)].

Factors	Total (*n* = 987)	SMS (*n* = 306)	Non-SMS (*n* = 681)	*χ^2^*/*Z*	*P*	Training set (*n* = 691)	Validation set (*n* = 296)
CRP	9.8 (0.9,7.8)	11.2 (1,8.5)	9.2 (0.9,7.3)	-0.731	0.4647	10 (1,8.1)	9.4 (0.9,6.3)
WBC	5.5 (3.9,6.5)	4.5 (3,5.4)	6 (4.5,6.7)	12.465	<0.001	5.5 (3.8,6.5)	5.6 (4.1,6.5)
Hemoglobin	119.4 (107,132.5)	116.7 (106,129)	120.6 (108,134)	3.234	0.001	119 (107,133)	120.3 (109,132)
PLT	198.1 (139,237)	173.4 (114,203.7)	209.2 (154,245)	7.331	<0.001	194.6 (135.5,235)	206.3 (148,246.3)
Albumin	39.6 (36.6,43.1)	38.9 (35.6,42.5)	40 (37.1,43.3)	3.574	<0.001	39.4 (36.1,43.1)	40.2 (37.6,43.1)
Creatinine	70.9 (57.4,82.0)	68.5 (53.9,80.6)	71.9 (58.4,82.1)	2.090	0.037	71.1 (57.3,82.1)	70.4 (57.6,80.6)
Glucose	6.0 (5.0,6.4)	6.1 (4.8,6.7)	6 (5.1,6.3)	1.254	0.210	6.1 (5,6.6)	5.9 (5.1,6.3)
Total bilirubin	13.9 (9.4,16.1)	14.3 (10.2,17.3)	13.8 (9.2,15.6)	-3.852	<0.001	13.9 (9.3,16.1)	14 (9.7,16.3)
D-dimer	4.5 (0.6,1.3)	4.1 (0.6,1.8)	4.7 (0.6,1.3)	0.723	0.470	3.6 (0.6,1.5)	6.6 (0.6,1.3)
ALT	24.1 (14.0,27.0)	23.4 (14,28)	24.4 (14,26)	-0.936	0.349	24.5 (14,28)	23 (14,26.3)
AST	29.4 (21.0,32.0)	29.6 (22,33)	29.3 (20,32)	-2.883	0.004	29.9 (21,32)	28.2 (20,31)
CEA	165.3 (2.6,46.3)	92.2 (2.3,48.7)	198.2 (2.3,42.1)	-3.205	0.001	138.6 (2.6,33.5)	227.8 (2.5,65.4)
Age	67.1 (64.0,73.0)	68.6 (64,74)	66.5 (64,72)	-2.742	0.006	67.4 (64,73)	66.4 (64,72)
Chemotherapy cycle	5.4 (2.0,7.0)	7 (4,9.8)	4.6 (2,6)	-8.763	<0.001	5.3 (2,7)	5.5 (2,8)
Male	759 (76.9%)	228 (74.5%)	531 (78%)	1.426	0.232	538 (77.9%)	221 (74.7%)
Right-side colon cancer	292 (29.6%)	96 (31.4%)	196 (28.8%)	0.681	0.409	208 (30.1%)	84 (28.4%)
Stage 4	453 (45.9%)	172 (56.2%)	281 (41.3%)	18.994	<0.001	313 (45.3%)	140 (47.3%)
Targeted therapy	468 (47.4%)	179 (58.5%)	289 (42.4%)	21.838	<0.001	325 (47%)	143 (48.3%)
KPS ≤70	288 (29.2%)	165 (53.9%)	123 (18.1%)	131.381	<0.001	203 (29.4%)	85 (28.7%)
Bone metastasis	102 (10.3%)	66 (21.6%)	36 (5.3%)	60.405	<0.001	67 (9.7%)	35 (11.8%)
Liver metastasis	249 (25.2%)	107 (35%)	142 (20.9%)	22.301	<0.001	179 (25.9%)	70 (23.6%)
Lung metastasis	181 (18.3%)	61 (19.9%)	120 (17.6%)	0.755	0.385	130 (18.8%)	51 (17.2%)
Radical surgery	892 (90.4%)	266 (86.9%)	626 (91.9%)	6.057	0.014	628 (90.9%)	264 (89.2%)
Smoking	245 (24.8%)	92 (30.1%)	153 (22.5%)	6.532	0.011	167 (24.2%)	78 (26.4%)
Diabetes mellitus	203 (20.6%)	85 (27.8%)	118 (17.3%)	14.113	<0.001	137 (19.8%)	66 (22.3%)
Hypertension	424 (43%)	129 (42.2%)	295 (43.3%)	0.116	0.733	302 (43.7%)	122 (41.2%)
RAS mutation	441 (44.7%)	145 (47.4%)	296 (43.5%)	1.313	0.252	303 (43.8%)	138 (46.6%)
BRAF mutation	144 (14.6%)	45 (14.7%)	99 (14.5%)	0.005	0.945	114 (16.5%)	30 (10.1%)
dMMR	117 (11.9%)	34 (11.1%)	83 (12.2%)	0.234	0.628	78 (11.3%)	39 (13.2%)

### 3.2 Results of LASSO regression on the training set

**Figure 1:**
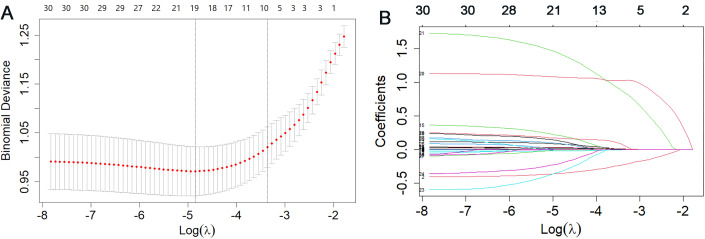
Variable selection based on LASSO regression on the training set. **(A)** Binomial deviance curve for predictor selection, **(B)** Coefficient paths of the predictors.

To select the optimal set of predictors, 29 potential influencing factors were introduced into the LASSO regression model (**Figure 1**). In **Figure 1A**, the dashed line ‘a’ represents the model selected at the minimum cross-validated devianc, while dashed line ‘b’ represents the model selected at one standard error above the minimum, which yields a model with good performance and the fewest predictors. **Figure 1B** shows the coefficient paths for each predictor as Log(λ) changes. As the λ value increased, the coefficients of most variables shrank towards zero. Nine variables with non-zero coefficients were retained and identified as predictors for SMS in the LASSO regression. The nine retained predictors were: CRP, WBC, PLT, age, number of chemotherapy cycles, KPS score, serum albumin, bone metastasis, and diabetes.

### 3.3 Development and selection of SMS prediction models

Based on the nine variables selected by LASSO regression, five models were constructed on the training set: Logistic regression, SVM, Decision Tree, XGBoost, and Random Forest. Their predictive performance was then evaluated on the validation set, as shown in **Table 2** and **Supplementary Figures 1** and **2**. Both the XGBoost and random forest models demonstrated strong discriminative performance, with AUC values of 0.906 and 0.905, respectively. Compared with the random forest model, the XGBoost model achieved higher sensitivity (0.864 vs. 0.716). Based on its overall performance metrics, the XGBoost model was selected as the final prediction model for subsequent interpretability analyses.

**Table 2:** Predictive performance of five machine-learning models in the validation set.

Model	Accuracy	Sensitivity	Specificity	F1	AUC
XGBoost	0.848	0.864	0.841	0.772	0.906
Random forest	0.875	0.716	0.942	0.773	0.905
Decision tree	0.848	0.796	0.870	0.757	0.839
SVM	0.787	0.648	0.846	0.644	0.810
Logistic regression	0.716	0.875	0.649	0.647	0.809

### 3.4 Global and individual interpretation of the XGBoost model

**Figure 2:**
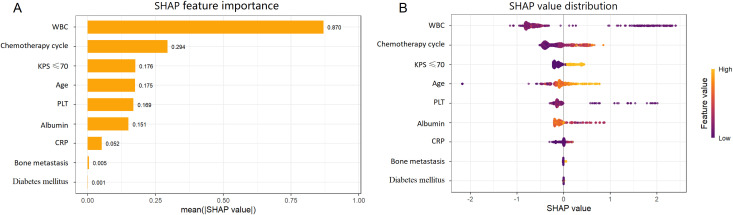
Global interpretability of the XGBoost model using SHAP. **(A)** Feature importance bar plot, **(B)** Beeswarm plot of feature contributions.

**Figure 3:**
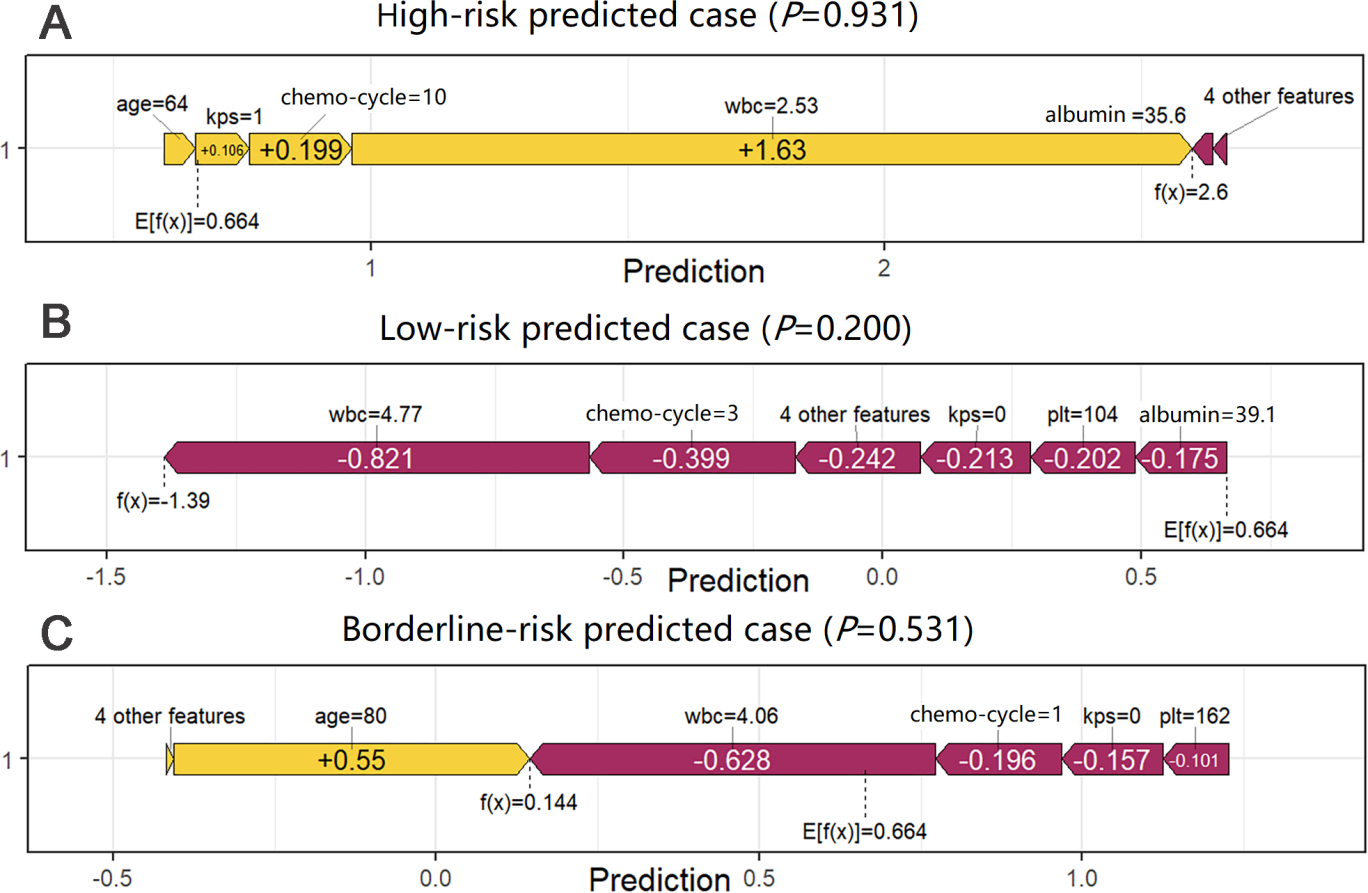
Waterfall plots for individual interpretation in the XGBoost model. **(A)** High-risk predicted case (ID 5), **(B)** Low-risk predicted case (ID 243), **(C)** Borderline-risk predicted case (ID 1).

Global and individual interpretability of the XGBoost model was assessed using SHAP. **Figure 2A** presents the nine predictors ranked by their mean absolute SHAP values: WBC, number of chemotherapy cycles, KPS score, age, PLT, serum albumin, CRP, bone metastasis, and diabetes. The SHAP beeswarm plot (**Figure 2B**) illustrates the distribution and direction of feature contributions. Lower pre-chemotherapy WBC and PLT values were associated with higher positive SHAP values, whereas higher values corresponded to negative SHAP values. Higher KPS scores and serum albumin levels were associated with negative SHAP values. In contrast, higher numbers of chemotherapy cycles, older age, higher CRP levels, and the presence of bone metastasis were associated with positive SHAP values. Diabetes showed a relatively smaller contribution, with SHAP values clustered near zero. Individual-level explanations were visualized using SHAP waterfall plots (**Figure 3**), illustrating the contribution of each feature to the predicted risk for representative high-risk (ID 5), low-risk (ID 243), and borderline-risk (ID 1) cases.

### 3.5 Nonlinear and interaction effects identified by SHAP analysis

**Figure 4:**
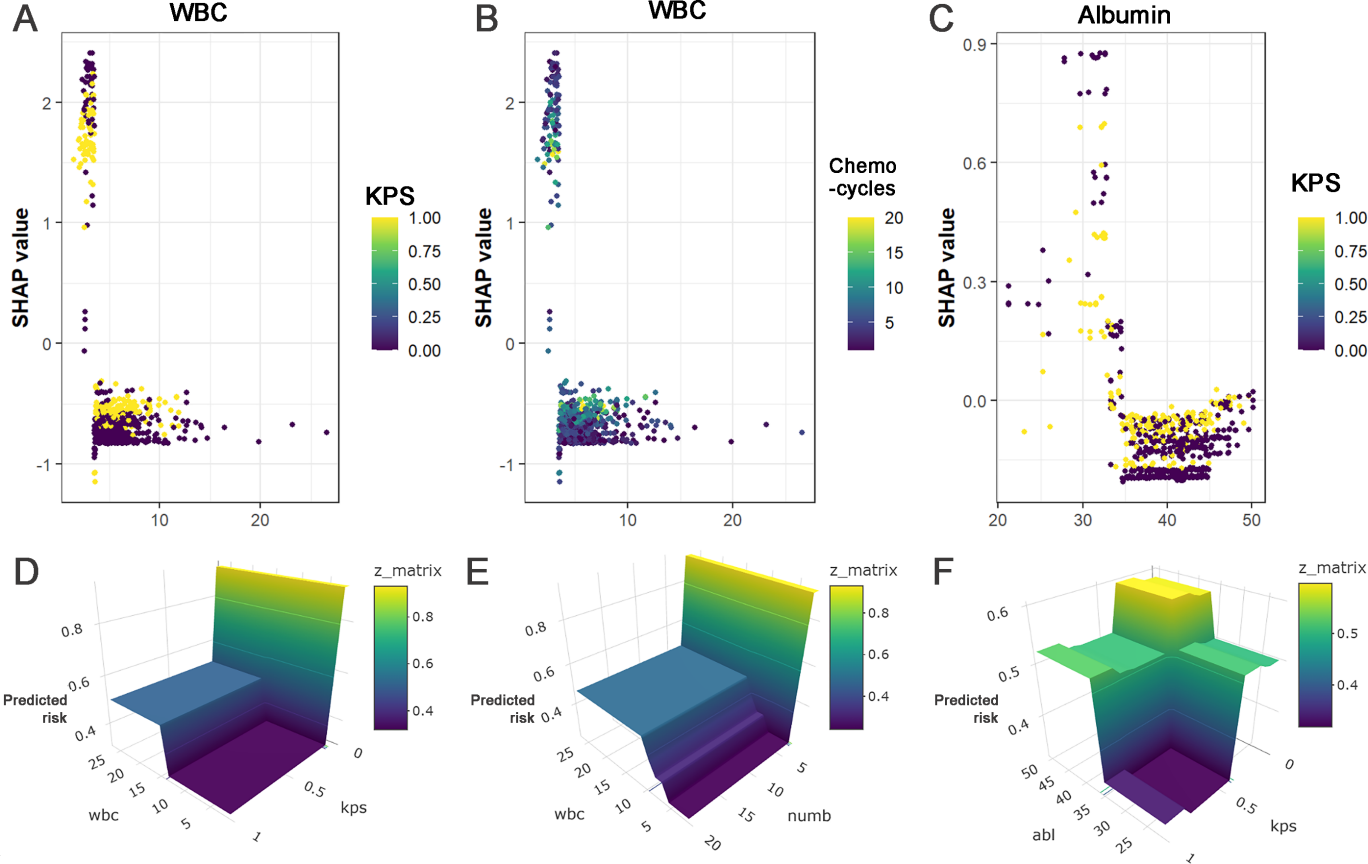
SHAP dependence plots and interaction surface plots. **(A)** SHAP dependence plot for WBC and KPS score, **(B)** SHAP dependence plot for WBC and number of chemotherapy cycles, **(C)** SHAP dependence plot for serum albumin and KPS score, **(D)** Interaction surface plot for WBC and KPS score, **(E)** Interaction surface plot for WBC and number of chemotherapy cycles, **(F)** Interaction surface plot for serum albumin and KPS score.

SHAP dependence plots (**Figures 4A–C**; **Supplementary Figure 3**) demonstrated nonlinear relationships between several continuous predictors and SMS risk. Notable threshold-like patterns were observed for pre-chemotherapy WBC, PLT, and serum albumin. SMS risk increased sharply when WBC fell below approximately 3.5×10^9^/L or PLT below approximately 80×10^9^/L, whereas risk changes were less pronounced above these levels. A similar pattern was observed for serum albumin around 35 g/L. CRP exhibited a different pattern, with higher SHAP values observed at levels above approximately 25 mg/L. SHAP interaction analyses revealed interactions between WBC and KPS score, WBC and number of chemotherapy cycles, and serum albumin and KPS score (**Figures 4D–F**). As shown in **Figures 4D**, the SHAP values associated with WBC varied across different KPS levels, with lower WBC values corresponding to higher positive SHAP values in patients with higher KPS scores, while higher WBC values were associated with more negative SHAP values. **Figure 4E** demonstrates an interaction between WBC and the number of chemotherapy cycles, in which the increase in SHAP values associated with decreasing WBC was more pronounced among patients with fewer chemotherapy cycles. In addition, **Figure 4F** shows an interaction between serum albumin and KPS score, whereby lower albumin levels were associated with higher positive SHAP values at higher KPS levels, whereas higher albumin levels corresponded to more negative SHAP values at lower KPS levels. Additional feature pairs showed weaker interaction effects (**Supplementary Figure 3**).

### 3.6 Development and validation of a web-based SMS risk calculator

**Figure 5:**
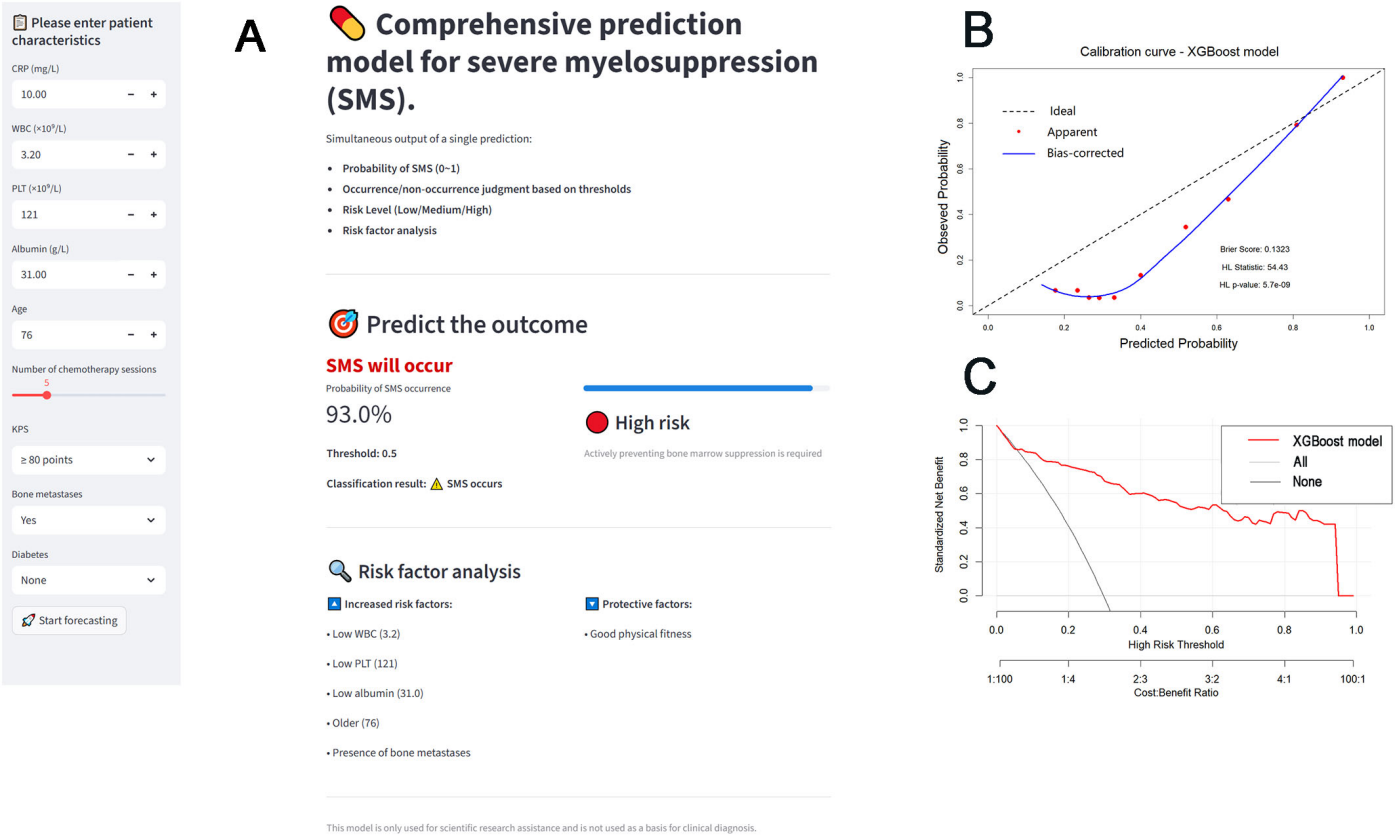
Clinical application and validation of the XGBoost-based SMS risk prediction model. **(A)** Interactive interface of the web-based SMS risk calculator, **(B)** Calibration curve of the XGBoost model on the validation set, **(C)** Decision curve analysis of the XGBoost model on the validation set.

A web-based SMS risk calculator was developed based on the trained XGBoost model (**Figure 5A**). The calculator allows users to input the nine predictors and returns the predicted probability and risk classification for SMS in real time. The complete Streamlit application and source code are provided as **Supplementary File 1**, enabling local deployment and reproduction of the web-based interface. Model calibration is shown in **Figure 5B**, demonstrating agreement between predicted and observed SMS probabilities. The Brier score was 0.116, further confirming the model’s satisfactory calibration. Decision curve analysis (**Figure 5C**) showed that the XGBoost-based strategy yielded a higher net benefit than “treat-all” or “treat-none” strategies across a range of threshold probabilities (0.1~0.9).

## 4 Discussion

This study developed and validated an XGBoost model, using an interpretable machine learning approach, to predict the risk of SMS in CRC patients receiving CapeOx ± Targ chemotherapy. By applying the SHAP interpretability framework, we quantified the contributions of nine predictors, including WBC, number of chemotherapy cycles, and KPS score, and revealed nonlinear threshold effects and interactions among them, which are insights often difficult to obtain with traditional linear models. In addition, the model was implemented as a web-based risk calculator, providing a practical framework for individualized SMS risk assessment in a clinical setting.

Previous studies have investigated chemotherapy-induced myelosuppression using traditional statistical models. For example, Wang et al. reported a logistic regression–based prediction model for myelosuppression in malignant lymphoma patients with an AUC of 0.861 (23), while a nomogram developed for nasopharyngeal carcinoma achieved an AUC of 0.744 (24). In colorectal cancer, a recent study reported an AUC of 0.80 for a logistic regression model predicting myelosuppression after first-line chemotherapy (25). Collectively, these findings highlight the performance limitations of traditional regression models for this task. More recently, machine learning approaches have been explored for predicting chemotherapy-related myelosuppression. Liu et al. compared multiple algorithms in CRC patients and reported superior performance for XGBoost (AUC = 0.97) (26), and Li et al. demonstrated favorable performance of an XGBoost-based model in lung cancer patients (AUC = 0.855) (27). In this context, the XGBoost model developed in the present study achieved an AUC of 0.906, with the additional advantage of interpretability through SHAP, enabling a more detailed characterization of nonlinear relationships and feature interactions.

The SHAP dependence plots in this study revealed patterns suggesting that the influence of continuous variables such as WBC, PLT, and serum albumin on SMS risk may exhibit distinct “threshold effects.” For example, the plots suggested that when pre-chemotherapy WBC fell below approximately 3.5×10^9^/L, PLT below approximately 80×10^9^/L, or albumin below approximately 35 g/L, SMS risk increased steeply with decreasing values; above these ranges, the risk increase plateaued, indicating the presence of risk inflection points and saturation periods. Although previous studies have noted associations between low WBC, low albumin, and myelosuppression risk (23, 25, 28–30), few have used interpretability techniques like SHAP to provide visualized, intuitive, and quantifiable risk thresholds for these continuous variables or to reveal the potential concept of a “plateau period.” From a pathophysiological perspective, these threshold-like patterns may reflect a “bone marrow reserve” phenomenon. Cytotoxic chemotherapy primarily targets rapidly proliferating hematopoietic progenitor cells, and pre-treatment blood counts reflect the residual marrow reserve following prior chemotherapy exposure and disease-related stress. Multiple studies have consistently identified low baseline blood cell counts as important and reproducible predictors of excessive hematologic toxicity in patients receiving chemotherapy (23, 28, 29). Reduced baseline WBC or PLT counts may indicate impaired granulopoiesis or megakaryopoiesis, incomplete hematologic recovery between cycles, or diminished compensatory capacity of the bone marrow. Therefore, initiating a new chemotherapy cycle when hematologic reserve is already near a critical low range may increase susceptibility to severe cytopenias.

Hypoalbuminemia represents a composite marker of systemic inflammation and nutritional vulnerability rather than solely a nutritional parameter. Both chronic inflammation and malnutrition may impair hematopoietic recovery, tissue repair, and physiological resilience following cytotoxic stress (31). In addition, serum albumin is a major drug-binding protein; decreased albumin levels may increase the free (unbound) fraction of certain agents, potentially altering pharmacokinetics and amplifying toxicity in susceptible individuals (32). The observed threshold around 35 g/L may therefore represent a clinically meaningful transition into a state of reduced physiological reserve, in which tolerance to chemotherapy-induced marrow injury is substantially diminished. These values may serve as clinically useful risk-alert thresholds, particularly for identifying patients in borderline states who are more sensitive to fluctuations in these parameters, thereby enabling enhanced monitoring and preemptive intervention. They also imply that in clinical practice, there may be no need to excessively pursue values far above the normal range for parameters like WBC, PLT, or serum albumin to obtain additional protection; maintaining levels above these thresholds may be sufficient for effective risk control. This provides a rationale for the judicious use of medications such as granulocyte colony-stimulating factor (G-CSF) and human albumin, potentially helping to avoid overtreatment.

Through SHAP interaction analysis, this study also systematically identified clinically meaningful interaction effects among different predictors. We found that the association between WBC and SMS risk does not exist in isolation but interacts with the patient’s performance status (KPS score) and the number of chemotherapy cycles. In patients with high KPS scores, low WBC was associated with a more pronounced increase in SMS risk; conversely, the inverse association between high WBC and lower risk was also stronger. This suggests that good performance status may amplify the sensitivity of bone marrow reserve function to chemotherapy toxicity. Similarly, in the early stages of chemotherapy with fewer cycles, a decrease in WBC could lead to a sharp increase in risk, highlighting the importance of close blood count monitoring during initial treatment. Furthermore, an interaction existed between serum albumin and KPS score: for patients with poor performance status, the association between higher albumin and lower SMS risk appeared more pronounced. Existing literature often discusses such factors as independent risk factors (23, 25, 28–30) or only presents their feature importance rankings. This study further utilized SHAP interaction plots to intuitively illustrate interaction effects between features, such as the complex combined effect pattern where “performance status may amplify the sensitivity of bone marrow reserve function.” These findings challenge the traditional view of risk factors acting independently and emphasize the importance of a comprehensive, dynamic assessment of patient risk, offering potential for more refined individualized risk management.

Most of the nine predictors ultimately included in our model have biological and clinical relevance supported by existing literature (3, 12–14, 25–27, 33, 34). Compared with traditional logistic regression, SHAP analysis provided a more intuitive and quantitative characterization of each feature’s contribution and direction of association through mean absolute SHAP value ranking, beeswarm plots, and dependence plots, rather than relying solely on odds ratios. Unlike some previous studies (13, 24, 25, 27), independent predictive value was not observed for factors such as sex, hemoglobin, bilirubin, tumor location, or body mass index in the present cohort, which may reflect differences in study populations, chemotherapy regimens, or outcome definitions.

To facilitate clinical application, a web-based, real-time SMS risk calculator was developed based on the XGBoost model. The tool allows clinicians to input nine variables and obtain individualized risk probabilities and risk classifications in real time. Compared with prediction models that remain limited to static nomograms or manual calculations (12, 13, 23–25), this calculator offers improved usability and automation. In addition, its decoupled RESTful API architecture provides flexibility for future functional expansion or potential integration with electronic medical record systems. Given that dedicated prediction tools for SMS following specific chemotherapy regimens in colorectal cancer—particularly those based on explainable machine learning—remain scarce, translating an interpretable machine learning model into a practical decision-support prototype represents a meaningful step toward individualized risk assessment.

Several limitations should be acknowledged. First, as a single-center retrospective study with only internal validation via random splitting, it is inevitably subject to selection bias and information bias, and external validation was not performed, which may limit the model’s external generalizability. Second, some potential influencing factors were not included, such as patient pharmacogenetic characteristics (e.g., DPYD genotype) (35), the actual dose-intensity curve of chemotherapy drugs, and detailed information on prophylactic medications (e.g., use of G-CSF). Third, while the identified nonlinear and interaction effects have biological plausibility and clinical relevance, their underlying biological mechanisms still require further validation through basic or prospective studies. Finally, the web-based calculator is currently a prototype and has not undergone prospective validation in real-world clinical settings. Future studies should involve multi-center cohorts, prospective external validation, and exploration of how nonlinear and interaction patterns can be translated into robust clinical decision-support strategies.

## 5 Conclusion

In summary, this study developed and preliminarily validated an XGBoost-based machine learning model for predicting SMS risk in colorectal cancer patients undergoing chemotherapy. Beyond demonstrating favorable predictive performance, the model leveraged SHAP interpretability to characterize nonlinear threshold patterns and interaction effects among risk factors, offering additional insight into the complex risk structure of chemotherapy-induced myelosuppression. By implementing a web-based calculator prototype, this study also explored a practical pathway for translating an explainable machine learning model into a clinically usable tool. Despite its limitations, the present work provides a methodological framework and a promising foundation for applying explainable machine learning to support more precise and individualized management of chemotherapy-related toxicity.

## Data Availability

The raw data supporting the conclusions of this article will be made available by the authors, without undue reservation.
