# Supplementary figures and images for: Interpretable machine-learning prediction of severe myelosuppression in colorectal cancer patients receiving chemotherapy using XGBoost and SHAP: a retrospective study with a web-based calculator
Source: Front Oncol. 2026 Mar 19;16:1785146. doi: 10.3389/fonc.2026.1785146 (PMC13043381; doi:10.3389/fonc.2026.1785146)

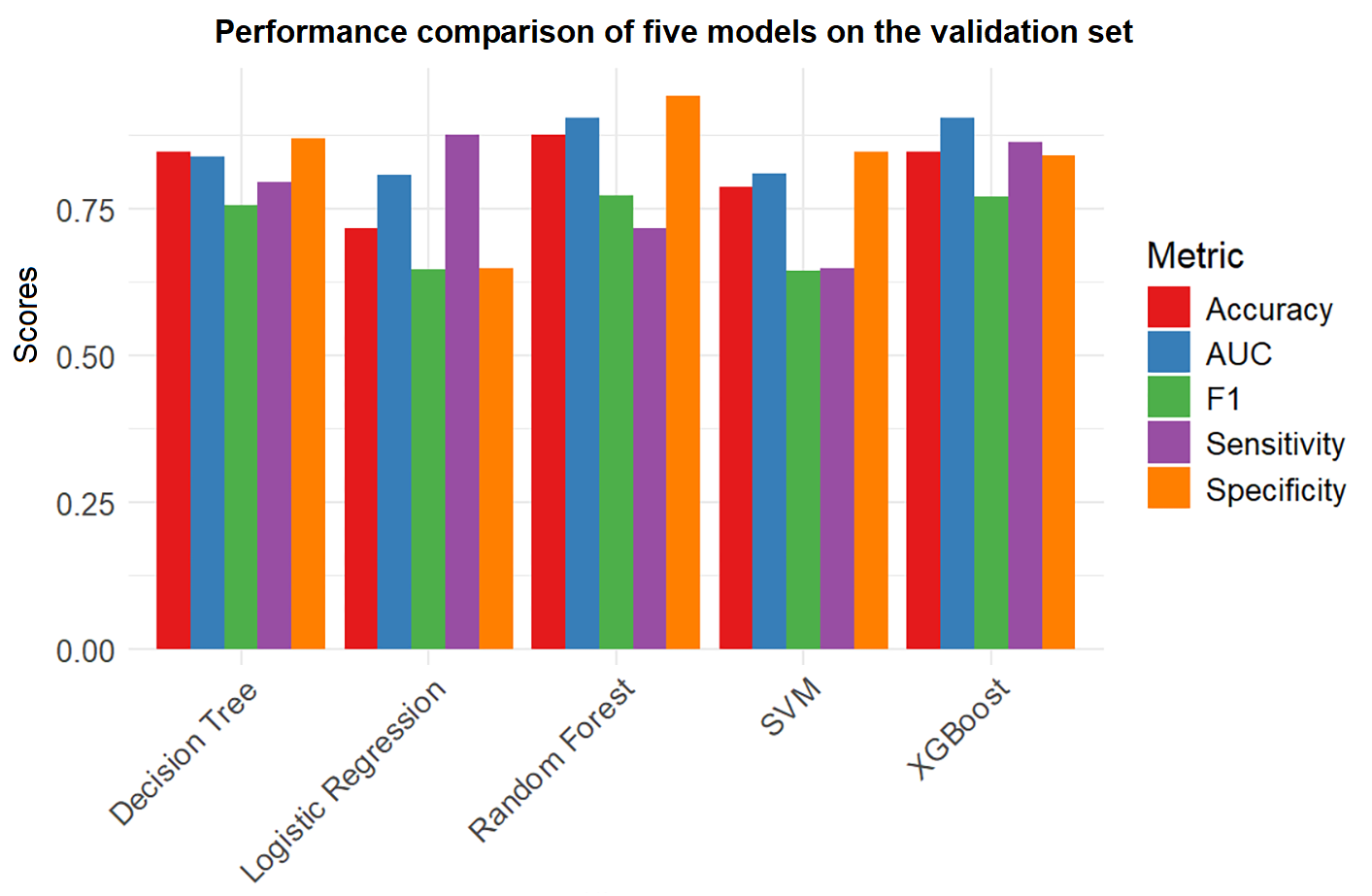

Supplement: Supplementary file 1 [file Image1.tiff]

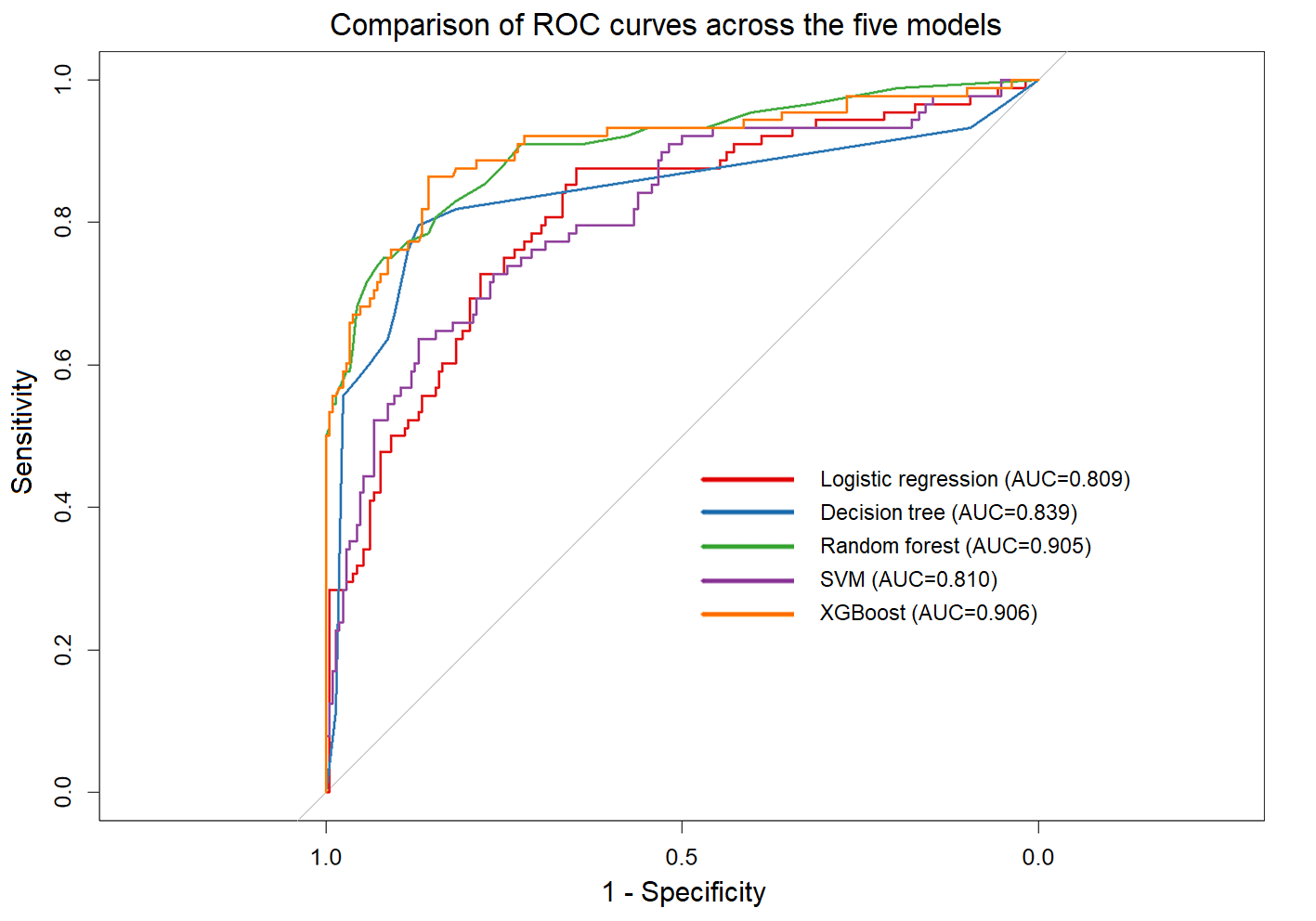

Supplement: Supplementary file 2 [file Image2.tif]

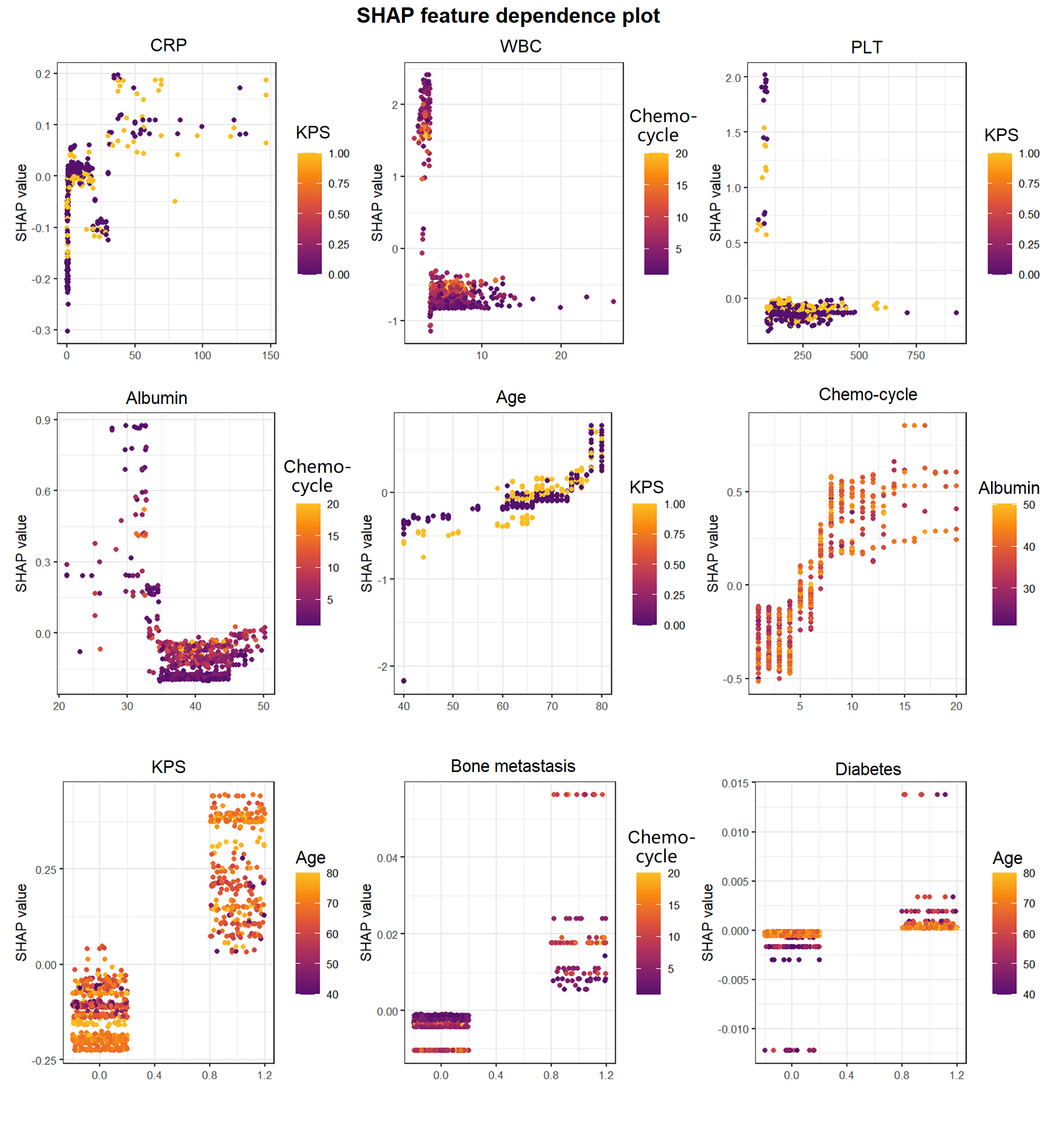

Supplement: Supplementary file 3 [file Image3.tif]
